# *n*-Butyldichlorido{4-cyclo­hexyl-1-[1-(pyridin-2-yl-κ*N*)ethyl­idene]thio­semi­carb­azi­dato-κ^2^ *N* ^1^,*S*}tin(IV)

**DOI:** 10.1107/S1600536812025937

**Published:** 2012-06-16

**Authors:** Md. Abu Affan, Md. Abdus Salam, Mohd Razip Asaruddin, Seik Weng Ng, Edward R. T. Tiekink

**Affiliations:** a Faculty of Resource Science and Technology, Universiti Malaysia Sarawak, 94300 Kota Samaharan, Sawarak, Malaysia; b Department of Chemistry, University of Malaya, 50603 Kuala Lumpur, Malaysia; c Chemistry Department, Faculty of Science, King Abdulaziz University, PO Box 80203 Jeddah, Saudi Arabia

## Abstract

Two independent mol­ecules comprise the asymmetric unit in the title compound, [Sn(C_4_H_9_)(C_14_H_19_N_4_S)Cl_2_]. In each mol­ecule, the Sn^IV^ atom exists within a distorted octa­hedral geometry defined by the *N*,*N*′,*S*-tridentate mono-deprotonated Schiff base ligand, two mutually *trans* Cl atoms, and the α-C atom of the *n*-butyl group; the latter is *trans* to the azo-N atom. The greatest distortion from the ideal geometry is found in the nominally *trans* angle formed by the S and pyridyl-N atoms at Sn [151.72 (7) and 152.04 (7)°, respectively]. In the crystal, mol­ecules are consolidated into a three-dimensional architecture by a combination of N—H⋯Cl, C—H⋯π and π–π inter­actions [inter-centroid distances = 3.6718 (19) and 3.675 (2) Å].

## Related literature

For the structures of the methyl­tin and phenyl­tin derivatives, see: Salam *et al.* (2010*a* ▶,*b* ▶).
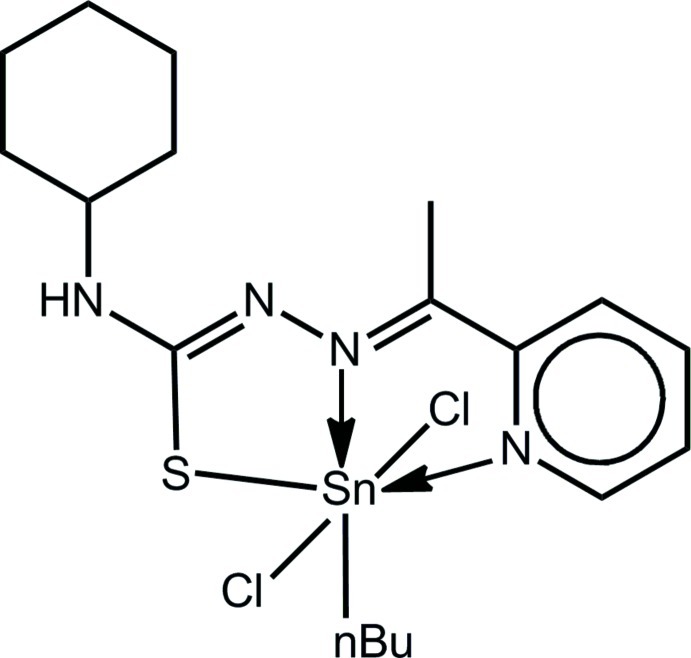


## Experimental

### 

#### Crystal data

[Sn(C_4_H_9_)(C_14_H_19_N_4_S)Cl_2_]
*M* *_r_* = 522.09
Monoclinic, 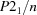

*a* = 12.1229 (3) Å
*b* = 15.4518 (4) Å
*c* = 23.6868 (6) Å
β = 103.894 (3)°
*V* = 4307.21 (19) Å^3^
*Z* = 8
Mo *K*α radiation
μ = 1.54 mm^−1^
*T* = 100 K
0.25 × 0.25 × 0.25 mm

#### Data collection

Agilent SuperNova Dual diffractometer with Atlas detector
Absorption correction: multi-scan (*CrysAlis PRO*; Agilent, 2012 ▶) *T*_min_ = 0.794, *T*_max_ = 1.000
18205 measured reflections
9861 independent reflections
8503 reflections with *I* > 2σ(*I*)
*R*_int_ = 0.024

#### Refinement

*R*[*F* ^2^ > 2σ(*F* ^2^)] = 0.035
*wR*(*F* ^2^) = 0.085
*S* = 1.04
9860 reflections
471 parameters
H-atom parameters constrained
Δρ_max_ = 1.64 e Å^−3^
Δρ_min_ = −1.11 e Å^−3^

### 

Data collection: *CrysAlis PRO* (Agilent, 2012 ▶); cell refinement: *CrysAlis PRO*; data reduction: *CrysAlis PRO*; program(s) used to solve structure: *SHELXS97* (Sheldrick, 2008 ▶); program(s) used to refine structure: *SHELXL97* (Sheldrick, 2008 ▶); molecular graphics: *ORTEP-3* (Farrugia, 1997 ▶), *QMol* (Gans & Shalloway, 2001 ▶) and *DIAMOND* (Brandenburg, 2006 ▶); software used to prepare material for publication: *publCIF* (Westrip, 2010 ▶).

## Supplementary Material

Crystal structure: contains datablock(s) global, I. DOI: 10.1107/S1600536812025937/qm2072sup1.cif

Structure factors: contains datablock(s) I. DOI: 10.1107/S1600536812025937/qm2072Isup2.hkl

Additional supplementary materials:  crystallographic information; 3D view; checkCIF report

## Figures and Tables

**Table 1:** Selected bond lengths (Å)

Sn1—C1	2.187 (3)
Sn1—N1	2.269 (2)
Sn1—N2	2.209 (2)
Sn1—S1	2.4785 (8)
Sn1—Cl1	2.5123 (8)
Sn1—Cl2	2.4959 (8)
Sn2—C19	2.182 (3)
Sn2—N5	2.255 (3)
Sn2—N6	2.215 (3)
Sn2—S2	2.4806 (8)
Sn2—Cl3	2.4959 (8)
Sn2—Cl4	2.5124 (8)

**Table 2:** Hydrogen-bond geometry (Å, °) *Cg*1 is the centroid of the N1,C5–C9 ring.

*D*—H⋯*A*	*D*—H	H⋯*A*	*D*⋯*A*	*D*—H⋯*A*
N4—H4⋯Cl3	0.88	2.65	3.516 (3)	167
C15—H15*A*⋯*Cg*1^i^	0.99	2.85	3.692 (4)	143

Symmetry code: (i) 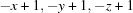.

**Fig. 1.**
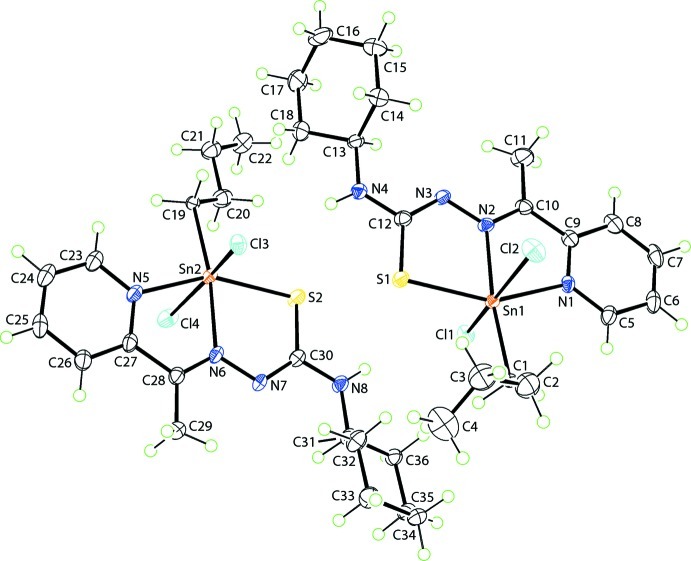
The molecular structures of the two independent molecules of (I) showing the atom-labelling scheme and displacement ellipsoids at the 50% probability level.

**Fig. 2.**
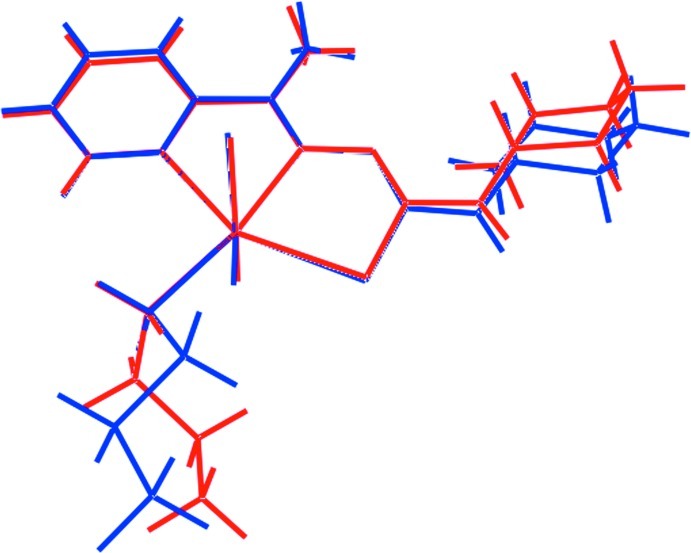
Superimposition of the two independent molecules in (I). The S,N-chelate rings have been superimposed, and the Sn1 and and Sn2-containing molecules are shown as red and blue images, respectively.

**Fig. 3.**
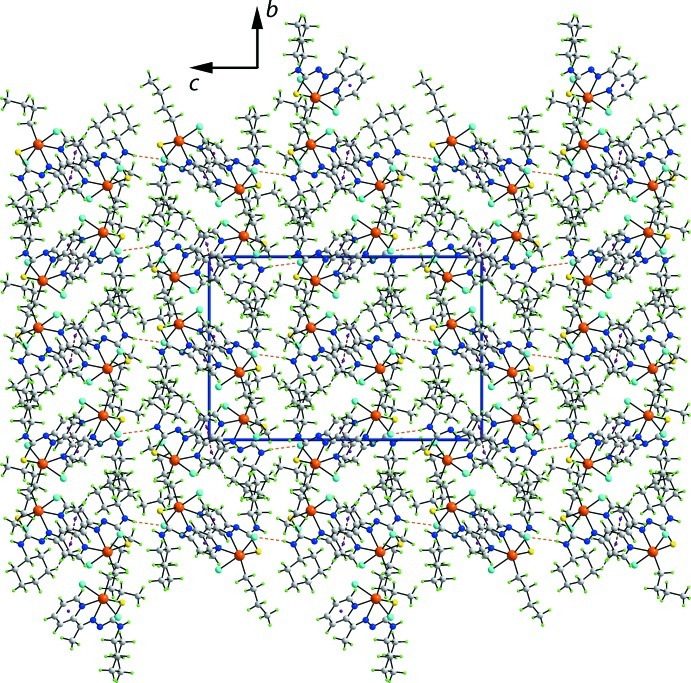
A view in projection down the a axis of the unit-cell contents for (I). The N—H···Cl, C—H···π and π—π interactions are shown as orange, purple and brown dashed lines, respectively.

## supplementary crystallographic information

### Comment

Previous structural studies have described the methyltin (Salam *et al.*, 2010*a*) and phenyltin (Salam *et al.*, 2010*b*) derivatives of the title compound. The molecular structure of the title compound, (I), resembles these.

There are two independent molecules in the asymmetric unit of (I), Fig. 1. These differ in terms of the relative dispositions of the *n*-butyl and cycohexyl rings, Fig. 2. The Sn atom in each molecule exists within a six atom CCl_2_N_2_S donor set defined by the tridentate mono-deprotonated Schiff base ligand, two mutually *trans* chlorido atoms, and the α-C atom of the Sn-bound *n*-butyl group which is *trans* to the azo-N atom, Table 1. Distortions from the ideal octahedral geometry are ascribed primarily to the restricted bite distances formed by the Schiff base which results in an angle of 151.72 (7) ° [152.04 (7)° for the second molecule] for the nominally *trans* S—Sn—N angle.

The molecules are consolidated into a three-dimensional architecture by a combination of N—H···Cl and C—H···π, Table 1, as well as π—π interactions, the latter occurring between centrosymmetrically related pairs of (N1,C5–C9) and (N5,C23–C27) rings [inter-centroid distances = 3.6718 (19) and 3.675 (2) Å for symmetry operations: 2 - *x*, 1 - *y*, 1 - *z* and -*x*, 1 - *y*, -*z*, respectively], Fig. 3 and Table 2.

### Experimental

2-Acetylpyridine-*N*(4)-cyclohexylthiosemicarbazone (0.28 g, 1.0 mmol) was dissolved in absolute methanol (10 ml) in a Schlenk round bottom flask under a nitrogen atmosphere. Then, a 10 ml me thanolic solution of butyltin(IV) trichloride (0.282 g, 1.0 mmol) was added drop-wise while stirring which resulted in the formation of a yellow solution. The reaction mixture was refluxed for 4 h and then cooled to room temperature. The yellow microcrystals that formed were filtered off, washed with a small amount of cold methanol and dried *in vacuo* over silica gel. Yellow crystals suitable for X-ray diffraction were obtained from the slow evaporation of a chloroform/methanol (1:1 ratio) solution at room temperature. Yield: 0.438 g, 78%: *M*.pt: 521–523 K: FT—IR (KBr, cm^-1^) ν_max_: 3308 (s, NH), 2931, 2855 (s, cyclohexyl), 1602 (m, C═N—N═C), 1020 (w, N—N), 1345, 833 (m, C—S), 652 (w, pyridine in plane), 570 (w, Sn—C), 475 (w, Sn—N). Anal. Calc. for C_18_H_28_Cl_2_N_4_SSn: C, 41.40; H, 5.40; N, 10.73%. Found: C, 41.24; H, 5.17; N, 10.59%.

### Refinement

Carbon-bound H-atoms were placed in calculated positions [C–H 0.95 to 0.98 Å, *U*_iso_(H) 1.2 to 1.5*U*_eq_(C)] and were included in the refinement in the riding model approximation. The amino H-atoms were similarly treated [N–H 0.88 Å; *U*_iso_(H) 1.2*U*_eq_(N)]. The (0 1 2) reflection was omitted from the final refinement as it was affected by the beam-stop. The maximum and minimum residual electron density peaks of 1.64 and 1.11 e Å^-3^, respectively, were located 0.73 Å and 0.74 Å from the Sn1 and Sn2 atoms, respectively.

### Crystal data

**Table:**

[Sn(C_4_H_9_)(C_14_H_19_N_4_S)Cl_2_]	*F*(000) = 2112
*M**_r_* = 522.09	*D*_x_ = 1.610 Mg m^−^^3^
Monoclinic, *P*2_1_/*n*	Mo *K*α radiation, λ = 0.71073 Å
Hall symbol: -P 2yn	Cell parameters from 10393 reflections
*a* = 12.1229 (3) Å	θ = 2.2–27.5°
*b* = 15.4518 (4) Å	µ = 1.54 mm^−^^1^
*c* = 23.6868 (6) Å	*T* = 100 K
β = 103.894 (3)°	Block, dark-yellow
*V* = 4307.21 (19) Å^3^	0.25 × 0.25 × 0.25 mm
*Z* = 8	

### Data collection

**Table:**

Agilent SuperNova Dual diffractometer with Atlas detector	9861 independent reflections
Radiation source: SuperNova (Mo) X-ray Source	8503 reflections with *I* > 2σ(*I*)
Mirror monochromator	*R*_int_ = 0.024
Detector resolution: 10.4041 pixels mm^-1^	θ_max_ = 27.6°, θ_min_ = 2.2°
ω scan	*h* = −15→11
Absorption correction: multi-scan (*CrysAlis PRO*; Agilent, 2012)	*k* = −14→19
*T*_min_ = 0.794, *T*_max_ = 1.000	*l* = −21→30
18205 measured reflections	

### Refinement

**Table:**

Refinement on *F*^2^	Primary atom site location: structure-invariant direct methods
Least-squares matrix: full	Secondary atom site location: difference Fourier map
*R*[*F*^2^ > 2σ(*F*^2^)] = 0.035	Hydrogen site location: inferred from neighbouring sites
*wR*(*F*^2^) = 0.085	H-atom parameters constrained
*S* = 1.04	*w* = 1/[σ^2^(*F*_o_^2^) + (0.0351*P*)^2^ + 4.9209*P*] where *P* = (*F*_o_^2^ + 2*F*_c_^2^)/3
9860 reflections	(Δ/σ)_max_ = 0.001
471 parameters	Δρ_max_ = 1.64 e Å^−^^3^
0 restraints	Δρ_min_ = −1.11 e Å^−^^3^

### Fractional atomic coordinates and isotropic or equivalent isotropic displacement parameters (Å^2^)

**Table:**

	*x*	*y*	*z*	*U*_iso_*/*U*_eq_	
Sn1	0.671827 (17)	0.387870 (13)	0.373102 (9)	0.01486 (6)	
Sn2	0.329033 (17)	0.631862 (13)	0.111105 (9)	0.01785 (6)	
Cl1	0.79254 (6)	0.48374 (5)	0.32677 (3)	0.02156 (16)	
Cl2	0.56865 (7)	0.31624 (6)	0.43974 (4)	0.03090 (19)	
Cl3	0.23119 (6)	0.53083 (5)	0.16539 (3)	0.02212 (16)	
Cl4	0.40300 (7)	0.71030 (5)	0.03486 (4)	0.02837 (19)	
S1	0.49840 (7)	0.42958 (5)	0.29902 (3)	0.02126 (17)	
S2	0.51623 (7)	0.61085 (5)	0.18026 (4)	0.02298 (17)	
N1	0.8063 (2)	0.41305 (16)	0.45630 (11)	0.0172 (5)	
N2	0.6262 (2)	0.50862 (16)	0.41198 (11)	0.0168 (5)	
N3	0.5324 (2)	0.55449 (16)	0.38600 (11)	0.0193 (5)	
N4	0.3817 (2)	0.57067 (17)	0.30944 (12)	0.0203 (6)	
H4	0.3393	0.5528	0.2759	0.024*	
N5	0.1915 (2)	0.59176 (17)	0.03328 (11)	0.0197 (6)	
N6	0.3911 (2)	0.51522 (16)	0.07398 (11)	0.0187 (5)	
N7	0.4932 (2)	0.47828 (17)	0.09891 (11)	0.0199 (6)	
N8	0.6517 (2)	0.48053 (17)	0.17334 (12)	0.0210 (6)	
H8	0.6922	0.5051	0.2051	0.025*	
C1	0.7438 (3)	0.27114 (19)	0.34395 (13)	0.0203 (7)	
H1A	0.7346	0.2748	0.3013	0.024*	
H1B	0.8263	0.2696	0.3622	0.024*	
C2	0.6913 (4)	0.1875 (3)	0.35779 (19)	0.0419 (10)	
H2A	0.6899	0.1882	0.3994	0.050*	
H2B	0.7410	0.1391	0.3521	0.050*	
C3	0.5712 (4)	0.1691 (3)	0.3220 (2)	0.0464 (10)	
H3A	0.5385	0.1208	0.3401	0.056*	
H3B	0.5233	0.2209	0.3224	0.056*	
C4	0.5687 (4)	0.1462 (3)	0.2607 (2)	0.0563 (13)	
H4A	0.4902	0.1346	0.2395	0.084*	
H4B	0.6152	0.0945	0.2600	0.084*	
H4C	0.5991	0.1944	0.2422	0.084*	
C5	0.8962 (3)	0.3615 (2)	0.47722 (15)	0.0233 (7)	
H5	0.9074	0.3121	0.4554	0.028*	
C6	0.9726 (3)	0.3781 (2)	0.52955 (15)	0.0281 (8)	
H6	1.0352	0.3405	0.5436	0.034*	
C7	0.9569 (3)	0.4498 (3)	0.56083 (15)	0.0299 (8)	
H7	1.0086	0.4624	0.5969	0.036*	
C8	0.8641 (3)	0.5040 (2)	0.53929 (14)	0.0259 (7)	
H8A	0.8521	0.5540	0.5604	0.031*	
C9	0.7897 (2)	0.4837 (2)	0.48645 (13)	0.0195 (6)	
C10	0.6895 (3)	0.5371 (2)	0.46050 (14)	0.0204 (6)	
C11	0.6633 (3)	0.6176 (2)	0.48942 (17)	0.0341 (9)	
H11A	0.6054	0.6510	0.4621	0.051*	
H11B	0.7325	0.6524	0.5017	0.051*	
H11C	0.6348	0.6024	0.5235	0.051*	
C12	0.4730 (3)	0.5236 (2)	0.33567 (14)	0.0194 (6)	
C13	0.3504 (3)	0.65124 (19)	0.33522 (13)	0.0184 (6)	
H13	0.4217	0.6840	0.3525	0.022*	
C14	0.2903 (3)	0.6323 (2)	0.38348 (15)	0.0235 (7)	
H14A	0.3404	0.5971	0.4141	0.028*	
H14B	0.2201	0.5989	0.3676	0.028*	
C15	0.2607 (3)	0.7172 (2)	0.40959 (16)	0.0299 (8)	
H15A	0.2189	0.7045	0.4398	0.036*	
H15B	0.3315	0.7481	0.4284	0.036*	
C16	0.1885 (3)	0.7743 (2)	0.36318 (18)	0.0366 (9)	
H16A	0.1747	0.8302	0.3807	0.044*	
H16B	0.1140	0.7461	0.3477	0.044*	
C17	0.2450 (4)	0.7909 (2)	0.31372 (17)	0.0398 (10)	
H17A	0.3141	0.8262	0.3281	0.048*	
H17B	0.1925	0.8242	0.2829	0.048*	
C18	0.2775 (3)	0.7063 (2)	0.28791 (15)	0.0305 (8)	
H18A	0.2078	0.6740	0.2690	0.037*	
H18B	0.3197	0.7195	0.2580	0.037*	
C19	0.2561 (3)	0.75131 (18)	0.13533 (13)	0.0159 (6)	
H19A	0.2061	0.7781	0.1005	0.019*	
H19B	0.2100	0.7386	0.1636	0.019*	
C20	0.3533 (3)	0.8137 (2)	0.16256 (16)	0.0300 (8)	
H20A	0.4028	0.7866	0.1974	0.036*	
H20B	0.3999	0.8253	0.1343	0.036*	
C21	0.3060 (3)	0.8991 (2)	0.17965 (16)	0.0300 (8)	
H21A	0.2638	0.9291	0.1441	0.036*	
H21B	0.2519	0.8866	0.2040	0.036*	
C22	0.3993 (3)	0.9581 (2)	0.21298 (17)	0.0344 (8)	
H22A	0.3655	1.0118	0.2232	0.052*	
H22B	0.4520	0.9717	0.1887	0.052*	
H22C	0.4405	0.9290	0.2486	0.052*	
C23	0.0914 (3)	0.6321 (2)	0.01451 (15)	0.0251 (7)	
H23	0.0738	0.6799	0.0360	0.030*	
C24	0.0132 (3)	0.6060 (2)	−0.03525 (16)	0.0277 (8)	
H24	−0.0574	0.6352	−0.0476	0.033*	
C25	0.0392 (3)	0.5370 (2)	−0.06677 (15)	0.0271 (7)	
H25	−0.0130	0.5185	−0.1013	0.032*	
C26	0.1429 (3)	0.4948 (2)	−0.04721 (14)	0.0251 (7)	
H26	0.1618	0.4469	−0.0681	0.030*	
C27	0.2181 (3)	0.5233 (2)	0.00294 (14)	0.0206 (7)	
C28	0.3287 (3)	0.4809 (2)	0.02717 (14)	0.0199 (6)	
C29	0.3627 (3)	0.4028 (2)	−0.00154 (15)	0.0276 (8)	
H29A	0.4358	0.3813	0.0215	0.041*	
H29B	0.3701	0.4182	−0.0406	0.041*	
H29C	0.3048	0.3578	−0.0044	0.041*	
C30	0.5512 (3)	0.51657 (19)	0.14719 (14)	0.0190 (6)	
C31	0.6976 (3)	0.4020 (2)	0.15170 (14)	0.0201 (6)	
H31	0.6894	0.4084	0.1089	0.024*	
C32	0.6353 (3)	0.3209 (2)	0.16215 (16)	0.0270 (7)	
H32A	0.5549	0.3247	0.1402	0.032*	
H32B	0.6367	0.3165	0.2040	0.032*	
C33	0.6892 (3)	0.2400 (2)	0.14331 (16)	0.0293 (8)	
H33A	0.6822	0.2421	0.1008	0.035*	
H33B	0.6481	0.1881	0.1518	0.035*	
C34	0.8146 (3)	0.2328 (2)	0.17482 (15)	0.0270 (7)	
H34A	0.8220	0.2263	0.2172	0.032*	
H34B	0.8485	0.1811	0.1609	0.032*	
C35	0.8768 (3)	0.3138 (2)	0.16309 (15)	0.0267 (7)	
H35A	0.8746	0.3170	0.1211	0.032*	
H35B	0.9575	0.3101	0.1847	0.032*	
C36	0.8240 (3)	0.3964 (2)	0.18138 (14)	0.0210 (7)	
H36A	0.8340	0.3966	0.2241	0.025*	
H36B	0.8637	0.4477	0.1708	0.025*	

### Atomic displacement parameters (Å^2^)

**Table:**

	*U*^11^	*U*^22^	*U*^33^	*U*^12^	*U*^13^	*U*^23^
Sn1	0.01286 (11)	0.01495 (11)	0.01646 (11)	0.00097 (8)	0.00287 (8)	−0.00118 (7)
Sn2	0.01488 (11)	0.01646 (11)	0.02229 (12)	0.00343 (8)	0.00465 (9)	0.00327 (8)
Cl1	0.0192 (4)	0.0265 (4)	0.0189 (4)	−0.0054 (3)	0.0042 (3)	−0.0003 (3)
Cl2	0.0308 (4)	0.0354 (5)	0.0290 (4)	−0.0129 (4)	0.0121 (4)	0.0003 (4)
Cl3	0.0215 (4)	0.0236 (4)	0.0203 (4)	−0.0032 (3)	0.0031 (3)	0.0034 (3)
Cl4	0.0283 (4)	0.0253 (4)	0.0356 (5)	0.0044 (3)	0.0158 (4)	0.0093 (3)
S1	0.0168 (4)	0.0223 (4)	0.0214 (4)	0.0031 (3)	−0.0019 (3)	−0.0079 (3)
S2	0.0177 (4)	0.0191 (4)	0.0297 (4)	0.0030 (3)	0.0008 (3)	−0.0036 (3)
N1	0.0133 (12)	0.0217 (13)	0.0157 (13)	−0.0007 (11)	0.0015 (10)	0.0028 (10)
N2	0.0129 (12)	0.0185 (12)	0.0189 (13)	0.0000 (10)	0.0038 (10)	−0.0034 (10)
N3	0.0151 (12)	0.0196 (13)	0.0217 (14)	0.0039 (11)	0.0013 (11)	−0.0052 (11)
N4	0.0175 (13)	0.0227 (13)	0.0194 (13)	0.0030 (11)	0.0017 (11)	−0.0040 (11)
N5	0.0158 (13)	0.0216 (13)	0.0223 (14)	0.0049 (11)	0.0056 (11)	0.0064 (11)
N6	0.0135 (12)	0.0210 (13)	0.0210 (14)	0.0042 (11)	0.0028 (10)	0.0051 (11)
N7	0.0141 (12)	0.0227 (13)	0.0213 (14)	0.0057 (11)	0.0011 (11)	0.0019 (11)
N8	0.0155 (13)	0.0215 (13)	0.0238 (14)	0.0017 (11)	0.0003 (11)	−0.0031 (11)
C1	0.0259 (16)	0.0191 (15)	0.0136 (14)	0.0113 (13)	0.0002 (12)	−0.0029 (12)
C2	0.049 (2)	0.033 (2)	0.042 (2)	0.0048 (19)	0.008 (2)	0.0017 (18)
C3	0.049 (3)	0.040 (2)	0.050 (3)	0.004 (2)	0.012 (2)	0.000 (2)
C4	0.066 (3)	0.053 (3)	0.050 (3)	−0.018 (3)	0.014 (2)	0.000 (2)
C5	0.0166 (15)	0.0300 (17)	0.0231 (17)	0.0027 (14)	0.0044 (13)	0.0078 (14)
C6	0.0181 (16)	0.042 (2)	0.0219 (17)	0.0015 (15)	0.0001 (14)	0.0104 (15)
C7	0.0177 (16)	0.052 (2)	0.0171 (16)	−0.0074 (16)	−0.0012 (13)	0.0048 (16)
C8	0.0215 (16)	0.0353 (19)	0.0213 (17)	−0.0068 (15)	0.0060 (14)	−0.0023 (14)
C9	0.0131 (14)	0.0276 (16)	0.0182 (15)	−0.0052 (13)	0.0042 (12)	−0.0012 (13)
C10	0.0168 (15)	0.0246 (16)	0.0199 (16)	−0.0013 (13)	0.0046 (13)	−0.0057 (13)
C11	0.0293 (19)	0.0327 (19)	0.036 (2)	0.0007 (16)	0.0001 (16)	−0.0191 (16)
C12	0.0167 (15)	0.0209 (15)	0.0214 (16)	0.0000 (13)	0.0059 (13)	−0.0010 (12)
C13	0.0176 (15)	0.0175 (14)	0.0206 (16)	0.0033 (12)	0.0058 (13)	−0.0018 (12)
C14	0.0244 (17)	0.0220 (16)	0.0251 (17)	−0.0034 (14)	0.0081 (14)	−0.0025 (13)
C15	0.0311 (19)	0.0296 (18)	0.032 (2)	−0.0049 (16)	0.0132 (16)	−0.0087 (15)
C16	0.0300 (19)	0.032 (2)	0.048 (2)	0.0100 (16)	0.0083 (18)	−0.0124 (17)
C17	0.055 (3)	0.0286 (19)	0.033 (2)	0.0195 (19)	0.0063 (19)	0.0075 (16)
C18	0.037 (2)	0.0295 (18)	0.0239 (18)	0.0106 (16)	0.0052 (15)	0.0042 (14)
C19	0.0216 (15)	0.0121 (13)	0.0146 (14)	0.0057 (12)	0.0055 (12)	0.0038 (11)
C20	0.0323 (19)	0.0256 (17)	0.034 (2)	0.0057 (15)	0.0128 (16)	0.0048 (15)
C21	0.0302 (19)	0.0277 (18)	0.034 (2)	0.0049 (15)	0.0106 (16)	0.0036 (15)
C22	0.0277 (19)	0.0325 (19)	0.042 (2)	0.0031 (16)	0.0075 (17)	0.0015 (17)
C23	0.0194 (16)	0.0274 (17)	0.0283 (18)	0.0043 (14)	0.0053 (14)	0.0093 (14)
C24	0.0179 (16)	0.0328 (19)	0.0320 (19)	0.0043 (15)	0.0053 (14)	0.0143 (15)
C25	0.0194 (16)	0.038 (2)	0.0210 (17)	−0.0028 (15)	−0.0005 (13)	0.0090 (15)
C26	0.0199 (16)	0.0331 (18)	0.0217 (17)	0.0007 (14)	0.0035 (13)	0.0039 (14)
C27	0.0168 (15)	0.0258 (16)	0.0189 (16)	0.0028 (13)	0.0036 (12)	0.0073 (13)
C28	0.0165 (15)	0.0238 (16)	0.0194 (16)	0.0019 (13)	0.0046 (12)	0.0034 (13)
C29	0.0232 (17)	0.0313 (18)	0.0267 (18)	0.0069 (15)	0.0027 (14)	−0.0046 (14)
C30	0.0157 (15)	0.0176 (15)	0.0236 (16)	0.0006 (12)	0.0045 (13)	0.0027 (12)
C31	0.0146 (15)	0.0217 (15)	0.0230 (16)	0.0027 (13)	0.0026 (13)	−0.0002 (13)
C32	0.0184 (16)	0.0232 (16)	0.038 (2)	−0.0010 (14)	0.0040 (15)	0.0029 (15)
C33	0.0301 (18)	0.0205 (16)	0.033 (2)	−0.0003 (15)	−0.0002 (16)	−0.0050 (14)
C34	0.0296 (18)	0.0220 (16)	0.0281 (18)	0.0083 (15)	0.0044 (15)	−0.0025 (14)
C35	0.0199 (16)	0.0303 (18)	0.0283 (18)	0.0063 (14)	0.0027 (14)	−0.0053 (14)
C36	0.0171 (15)	0.0213 (15)	0.0233 (16)	0.0006 (13)	0.0022 (13)	−0.0035 (13)

### Geometric parameters (Å, º)

**Table:**

Sn1—C1	2.187 (3)	C13—H13	1.0000
Sn1—N1	2.269 (2)	C14—C15	1.529 (4)
Sn1—N2	2.209 (2)	C14—H14A	0.9900
Sn1—S1	2.4785 (8)	C14—H14B	0.9900
Sn1—Cl1	2.5123 (8)	C15—C16	1.513 (5)
Sn1—Cl2	2.4959 (8)	C15—H15A	0.9900
Sn2—C19	2.182 (3)	C15—H15B	0.9900
Sn2—N5	2.255 (3)	C16—C17	1.514 (5)
Sn2—N6	2.215 (3)	C16—H16A	0.9900
Sn2—S2	2.4806 (8)	C16—H16B	0.9900
Sn2—Cl3	2.4959 (8)	C17—C18	1.535 (5)
Sn2—Cl4	2.5124 (8)	C17—H17A	0.9900
S1—C12	1.757 (3)	C17—H17B	0.9900
S2—C30	1.754 (3)	C18—H18A	0.9900
N1—C5	1.345 (4)	C18—H18B	0.9900
N1—C9	1.346 (4)	C19—C20	1.539 (5)
N2—C10	1.296 (4)	C19—H19A	0.9900
N2—N3	1.356 (3)	C19—H19B	0.9900
N3—C12	1.324 (4)	C20—C21	1.530 (5)
N4—C12	1.346 (4)	C20—H20A	0.9900
N4—C13	1.476 (4)	C20—H20B	0.9900
N4—H4	0.8800	C21—C22	1.518 (5)
N5—C23	1.342 (4)	C21—H21A	0.9900
N5—C27	1.360 (4)	C21—H21B	0.9900
N6—C28	1.296 (4)	C22—H22A	0.9800
N6—N7	1.363 (3)	C22—H22B	0.9800
N7—C30	1.329 (4)	C22—H22C	0.9800
N8—C30	1.347 (4)	C23—C24	1.383 (5)
N8—C31	1.477 (4)	C23—H23	0.9500
N8—H8	0.8800	C24—C25	1.381 (5)
C1—C2	1.511 (5)	C24—H24	0.9500
C1—H1A	0.9900	C25—C26	1.393 (5)
C1—H1B	0.9900	C25—H25	0.9500
C2—C3	1.525 (6)	C26—C27	1.385 (5)
C2—H2A	0.9900	C26—H26	0.9500
C2—H2B	0.9900	C27—C28	1.479 (4)
C3—C4	1.488 (6)	C28—C29	1.491 (4)
C3—H3A	0.9900	C29—H29A	0.9800
C3—H3B	0.9900	C29—H29B	0.9800
C4—H4A	0.9800	C29—H29C	0.9800
C4—H4B	0.9800	C31—C32	1.514 (4)
C4—H4C	0.9800	C31—C36	1.527 (4)
C5—C6	1.382 (5)	C31—H31	1.0000
C5—H5	0.9500	C32—C33	1.526 (5)
C6—C7	1.370 (5)	C32—H32A	0.9900
C6—H6	0.9500	C32—H32B	0.9900
C7—C8	1.397 (5)	C33—C34	1.529 (5)
C7—H7	0.9500	C33—H33A	0.9900
C8—C9	1.392 (4)	C33—H33B	0.9900
C8—H8A	0.9500	C34—C35	1.521 (5)
C9—C10	1.475 (4)	C34—H34A	0.9900
C10—C11	1.491 (4)	C34—H34B	0.9900
C11—H11A	0.9800	C35—C36	1.537 (4)
C11—H11B	0.9800	C35—H35A	0.9900
C11—H11C	0.9800	C35—H35B	0.9900
C13—C18	1.511 (4)	C36—H36A	0.9900
C13—C14	1.524 (4)	C36—H36B	0.9900
			
C1—Sn1—N2	170.84 (11)	C15—C14—H14B	109.7
C1—Sn1—N1	99.08 (10)	H14A—C14—H14B	108.2
N2—Sn1—N1	72.10 (9)	C16—C15—C14	110.9 (3)
C1—Sn1—S1	109.02 (8)	C16—C15—H15A	109.5
N2—Sn1—S1	79.68 (7)	C14—C15—H15A	109.5
N1—Sn1—S1	151.72 (7)	C16—C15—H15B	109.5
C1—Sn1—Cl2	97.62 (9)	C14—C15—H15B	109.5
N2—Sn1—Cl2	84.11 (7)	H15A—C15—H15B	108.0
N1—Sn1—Cl2	83.77 (7)	C15—C16—C17	111.8 (3)
S1—Sn1—Cl2	95.41 (3)	C15—C16—H16A	109.3
C1—Sn1—Cl1	91.72 (9)	C17—C16—H16A	109.3
N2—Sn1—Cl1	85.09 (7)	C15—C16—H16B	109.3
N1—Sn1—Cl1	84.60 (6)	C17—C16—H16B	109.3
S1—Sn1—Cl1	91.23 (3)	H16A—C16—H16B	107.9
Cl2—Sn1—Cl1	166.10 (3)	C16—C17—C18	111.8 (3)
C19—Sn2—N6	172.08 (10)	C16—C17—H17A	109.3
C19—Sn2—N5	100.43 (10)	C18—C17—H17A	109.3
N6—Sn2—N5	72.94 (9)	C16—C17—H17B	109.3
C19—Sn2—S2	107.53 (8)	C18—C17—H17B	109.3
N6—Sn2—S2	79.14 (7)	H17A—C17—H17B	107.9
N5—Sn2—S2	152.04 (7)	C13—C18—C17	110.1 (3)
C19—Sn2—Cl3	96.95 (8)	C13—C18—H18A	109.6
N6—Sn2—Cl3	86.80 (7)	C17—C18—H18A	109.6
N5—Sn2—Cl3	84.25 (7)	C13—C18—H18B	109.6
S2—Sn2—Cl3	92.90 (3)	C17—C18—H18B	109.6
C19—Sn2—Cl4	91.03 (8)	H18A—C18—H18B	108.1
N6—Sn2—Cl4	83.88 (7)	C20—C19—Sn2	108.84 (19)
N5—Sn2—Cl4	82.31 (7)	C20—C19—H19A	109.9
S2—Sn2—Cl4	96.31 (3)	Sn2—C19—H19A	109.9
Cl3—Sn2—Cl4	165.42 (3)	C20—C19—H19B	109.9
C12—S1—Sn1	94.85 (11)	Sn2—C19—H19B	109.9
C30—S2—Sn2	95.45 (11)	H19A—C19—H19B	108.3
C5—N1—C9	119.8 (3)	C21—C20—C19	110.7 (3)
C5—N1—Sn1	124.6 (2)	C21—C20—H20A	109.5
C9—N1—Sn1	115.58 (19)	C19—C20—H20A	109.5
C10—N2—N3	118.6 (3)	C21—C20—H20B	109.5
C10—N2—Sn1	120.2 (2)	C19—C20—H20B	109.5
N3—N2—Sn1	121.25 (18)	H20A—C20—H20B	108.1
C12—N3—N2	115.8 (2)	C22—C21—C20	112.1 (3)
C12—N4—C13	122.0 (3)	C22—C21—H21A	109.2
C12—N4—H4	119.0	C20—C21—H21A	109.2
C13—N4—H4	119.0	C22—C21—H21B	109.2
C23—N5—C27	119.8 (3)	C20—C21—H21B	109.2
C23—N5—Sn2	125.1 (2)	H21A—C21—H21B	107.9
C27—N5—Sn2	115.03 (19)	C21—C22—H22A	109.5
C28—N6—N7	118.8 (3)	C21—C22—H22B	109.5
C28—N6—Sn2	119.3 (2)	H22A—C22—H22B	109.5
N7—N6—Sn2	121.91 (19)	C21—C22—H22C	109.5
C30—N7—N6	115.1 (3)	H22A—C22—H22C	109.5
C30—N8—C31	123.7 (3)	H22B—C22—H22C	109.5
C30—N8—H8	118.2	N5—C23—C24	121.8 (3)
C31—N8—H8	118.2	N5—C23—H23	119.1
C2—C1—Sn1	114.6 (2)	C24—C23—H23	119.1
C2—C1—H1A	108.6	C25—C24—C23	119.2 (3)
Sn1—C1—H1A	108.6	C25—C24—H24	120.4
C2—C1—H1B	108.6	C23—C24—H24	120.4
Sn1—C1—H1B	108.6	C24—C25—C26	119.1 (3)
H1A—C1—H1B	107.6	C24—C25—H25	120.5
C1—C2—C3	115.7 (3)	C26—C25—H25	120.5
C1—C2—H2A	108.3	C27—C26—C25	119.6 (3)
C3—C2—H2A	108.3	C27—C26—H26	120.2
C1—C2—H2B	108.3	C25—C26—H26	120.2
C3—C2—H2B	108.3	N5—C27—C26	120.6 (3)
H2A—C2—H2B	107.4	N5—C27—C28	116.6 (3)
C4—C3—C2	112.4 (4)	C26—C27—C28	122.8 (3)
C4—C3—H3A	109.1	N6—C28—C27	116.0 (3)
C2—C3—H3A	109.1	N6—C28—C29	123.6 (3)
C4—C3—H3B	109.1	C27—C28—C29	120.4 (3)
C2—C3—H3B	109.1	C28—C29—H29A	109.5
H3A—C3—H3B	107.9	C28—C29—H29B	109.5
C3—C4—H4A	109.5	H29A—C29—H29B	109.5
C3—C4—H4B	109.5	C28—C29—H29C	109.5
H4A—C4—H4B	109.5	H29A—C29—H29C	109.5
C3—C4—H4C	109.5	H29B—C29—H29C	109.5
H4A—C4—H4C	109.5	N7—C30—N8	116.4 (3)
H4B—C4—H4C	109.5	N7—C30—S2	128.3 (2)
N1—C5—C6	122.0 (3)	N8—C30—S2	115.3 (2)
N1—C5—H5	119.0	N8—C31—C32	112.3 (3)
C6—C5—H5	119.0	N8—C31—C36	107.8 (2)
C7—C6—C5	119.0 (3)	C32—C31—C36	111.3 (3)
C7—C6—H6	120.5	N8—C31—H31	108.4
C5—C6—H6	120.5	C32—C31—H31	108.4
C6—C7—C8	119.4 (3)	C36—C31—H31	108.4
C6—C7—H7	120.3	C31—C32—C33	111.4 (3)
C8—C7—H7	120.3	C31—C32—H32A	109.3
C9—C8—C7	119.0 (3)	C33—C32—H32A	109.3
C9—C8—H8A	120.5	C31—C32—H32B	109.3
C7—C8—H8A	120.5	C33—C32—H32B	109.3
N1—C9—C8	120.8 (3)	H32A—C32—H32B	108.0
N1—C9—C10	116.5 (3)	C32—C33—C34	111.3 (3)
C8—C9—C10	122.7 (3)	C32—C33—H33A	109.4
N2—C10—C9	115.6 (3)	C34—C33—H33A	109.4
N2—C10—C11	123.0 (3)	C32—C33—H33B	109.4
C9—C10—C11	121.3 (3)	C34—C33—H33B	109.4
C10—C11—H11A	109.5	H33A—C33—H33B	108.0
C10—C11—H11B	109.5	C35—C34—C33	109.2 (3)
H11A—C11—H11B	109.5	C35—C34—H34A	109.8
C10—C11—H11C	109.5	C33—C34—H34A	109.8
H11A—C11—H11C	109.5	C35—C34—H34B	109.8
H11B—C11—H11C	109.5	C33—C34—H34B	109.8
N3—C12—N4	115.5 (3)	H34A—C34—H34B	108.3
N3—C12—S1	128.4 (2)	C34—C35—C36	112.0 (3)
N4—C12—S1	116.2 (2)	C34—C35—H35A	109.2
N4—C13—C18	109.3 (3)	C36—C35—H35A	109.2
N4—C13—C14	111.4 (3)	C34—C35—H35B	109.2
C18—C13—C14	111.4 (3)	C36—C35—H35B	109.2
N4—C13—H13	108.2	H35A—C35—H35B	107.9
C18—C13—H13	108.2	C31—C36—C35	110.9 (3)
C14—C13—H13	108.2	C31—C36—H36A	109.5
C13—C14—C15	109.9 (3)	C35—C36—H36A	109.5
C13—C14—H14A	109.7	C31—C36—H36B	109.5
C15—C14—H14A	109.7	C35—C36—H36B	109.5
C13—C14—H14B	109.7	H36A—C36—H36B	108.0
			
C1—Sn1—S1—C12	−177.39 (14)	C7—C8—C9—C10	179.8 (3)
N2—Sn1—S1—C12	−0.37 (12)	N3—N2—C10—C9	179.6 (2)
N1—Sn1—S1—C12	−4.29 (18)	Sn1—N2—C10—C9	−0.4 (4)
Cl2—Sn1—S1—C12	82.62 (11)	N3—N2—C10—C11	0.5 (5)
Cl1—Sn1—S1—C12	−85.15 (11)	Sn1—N2—C10—C11	−179.5 (3)
C19—Sn2—S2—C30	−177.45 (13)	N1—C9—C10—N2	1.9 (4)
N6—Sn2—S2—C30	−1.86 (12)	C8—C9—C10—N2	−178.1 (3)
N5—Sn2—S2—C30	1.13 (18)	N1—C9—C10—C11	−179.0 (3)
Cl3—Sn2—S2—C30	84.32 (11)	C8—C9—C10—C11	1.1 (5)
Cl4—Sn2—S2—C30	−84.36 (11)	N2—N3—C12—N4	−178.4 (2)
C1—Sn1—N1—C5	−3.2 (3)	N2—N3—C12—S1	1.6 (4)
N2—Sn1—N1—C5	179.3 (3)	C13—N4—C12—N3	0.2 (4)
S1—Sn1—N1—C5	−176.62 (18)	C13—N4—C12—S1	−179.8 (2)
Cl2—Sn1—N1—C5	93.5 (2)	Sn1—S1—C12—N3	−0.5 (3)
Cl1—Sn1—N1—C5	−94.1 (2)	Sn1—S1—C12—N4	179.5 (2)
C1—Sn1—N1—C9	179.1 (2)	C12—N4—C13—C18	157.8 (3)
N2—Sn1—N1—C9	1.6 (2)	C12—N4—C13—C14	−78.7 (4)
S1—Sn1—N1—C9	5.7 (3)	N4—C13—C14—C15	179.3 (3)
Cl2—Sn1—N1—C9	−84.2 (2)	C18—C13—C14—C15	−58.4 (4)
Cl1—Sn1—N1—C9	88.2 (2)	C13—C14—C15—C16	56.8 (4)
N1—Sn1—N2—C10	−0.6 (2)	C14—C15—C16—C17	−55.3 (4)
S1—Sn1—N2—C10	−178.7 (2)	C15—C16—C17—C18	54.3 (4)
Cl2—Sn1—N2—C10	84.7 (2)	N4—C13—C18—C17	−179.4 (3)
Cl1—Sn1—N2—C10	−86.5 (2)	C14—C13—C18—C17	57.1 (4)
N1—Sn1—N2—N3	179.4 (2)	C16—C17—C18—C13	−54.7 (4)
S1—Sn1—N2—N3	1.3 (2)	N5—Sn2—C19—C20	−148.9 (2)
Cl2—Sn1—N2—N3	−95.3 (2)	S2—Sn2—C19—C20	30.4 (2)
Cl1—Sn1—N2—N3	93.5 (2)	Cl3—Sn2—C19—C20	125.7 (2)
C10—N2—N3—C12	178.0 (3)	Cl4—Sn2—C19—C20	−66.6 (2)
Sn1—N2—N3—C12	−2.0 (4)	Sn2—C19—C20—C21	179.4 (2)
C19—Sn2—N5—C23	−5.0 (3)	C19—C20—C21—C22	173.3 (3)
N6—Sn2—N5—C23	179.5 (3)	C27—N5—C23—C24	0.2 (5)
S2—Sn2—N5—C23	176.41 (18)	Sn2—N5—C23—C24	177.0 (2)
Cl3—Sn2—N5—C23	91.1 (2)	N5—C23—C24—C25	−0.6 (5)
Cl4—Sn2—N5—C23	−94.6 (2)	C23—C24—C25—C26	0.8 (5)
C19—Sn2—N5—C27	172.0 (2)	C24—C25—C26—C27	−0.6 (5)
N6—Sn2—N5—C27	−3.5 (2)	C23—N5—C27—C26	0.0 (5)
S2—Sn2—N5—C27	−6.6 (3)	Sn2—N5—C27—C26	−177.1 (2)
Cl3—Sn2—N5—C27	−91.9 (2)	C23—N5—C27—C28	−178.5 (3)
Cl4—Sn2—N5—C27	82.4 (2)	Sn2—N5—C27—C28	4.3 (3)
N5—Sn2—N6—C28	2.4 (2)	C25—C26—C27—N5	0.2 (5)
S2—Sn2—N6—C28	−179.0 (2)	C25—C26—C27—C28	178.7 (3)
Cl3—Sn2—N6—C28	87.4 (2)	N7—N6—C28—C27	179.0 (3)
Cl4—Sn2—N6—C28	−81.4 (2)	Sn2—N6—C28—C27	−1.0 (4)
N5—Sn2—N6—N7	−177.6 (2)	N7—N6—C28—C29	−0.4 (5)
S2—Sn2—N6—N7	0.9 (2)	Sn2—N6—C28—C29	179.6 (2)
Cl3—Sn2—N6—N7	−92.6 (2)	N5—C27—C28—N6	−2.3 (4)
Cl4—Sn2—N6—N7	98.6 (2)	C26—C27—C28—N6	179.2 (3)
C28—N6—N7—C30	−179.1 (3)	N5—C27—C28—C29	177.1 (3)
Sn2—N6—N7—C30	1.0 (4)	C26—C27—C28—C29	−1.4 (5)
N1—Sn1—C1—C2	99.3 (3)	N6—N7—C30—N8	178.9 (3)
S1—Sn1—C1—C2	−84.0 (2)	N6—N7—C30—S2	−3.6 (4)
Cl2—Sn1—C1—C2	14.4 (3)	C31—N8—C30—N7	−0.9 (4)
Cl1—Sn1—C1—C2	−175.9 (2)	C31—N8—C30—S2	−178.8 (2)
Sn1—C1—C2—C3	72.2 (4)	Sn2—S2—C30—N7	3.7 (3)
C1—C2—C3—C4	71.6 (5)	Sn2—S2—C30—N8	−178.7 (2)
C9—N1—C5—C6	0.6 (5)	C30—N8—C31—C32	−73.1 (4)
Sn1—N1—C5—C6	−177.0 (2)	C30—N8—C31—C36	164.0 (3)
N1—C5—C6—C7	−0.5 (5)	N8—C31—C32—C33	−176.1 (3)
C5—C6—C7—C8	0.1 (5)	C36—C31—C32—C33	−55.1 (4)
C6—C7—C8—C9	0.2 (5)	C31—C32—C33—C34	57.3 (4)
C5—N1—C9—C8	−0.3 (4)	C32—C33—C34—C35	−57.4 (4)
Sn1—N1—C9—C8	177.6 (2)	C33—C34—C35—C36	56.8 (4)
C5—N1—C9—C10	179.8 (3)	N8—C31—C36—C35	177.4 (3)
Sn1—N1—C9—C10	−2.4 (3)	C32—C31—C36—C35	53.9 (4)
C7—C8—C9—N1	−0.1 (5)	C34—C35—C36—C31	−55.6 (4)

### Hydrogen-bond geometry (Å, º)

Cg1 is the centroid of the N1,C5–C9 ring.

**Table:**

*D*—H···*A*	*D*—H	H···*A*	*D*···*A*	*D*—H···*A*
N4—H4···Cl3	0.88	2.65	3.516 (3)	167
C15—H15*A*···*Cg*1^i^	0.99	2.85	3.692 (4)	143

Symmetry code: (i) −*x*+1, −*y*+1, −*z*+1.
